# Community experiences of the impacts of climate-induced extreme weather events in Uganda: A qualitative study

**DOI:** 10.1371/journal.pgph.0005887

**Published:** 2026-03-20

**Authors:** Rawlance Ndejjo, Susan Karuhanga, Daniel Helldén, Idil Shekh Mohamed, Robert Marten, Tobias Alfvén, Rhoda K. Wanyenze

**Affiliations:** 1 Department of Disease Control and Environmental Health, School of Public Health, Makerere University, Kampala, Uganda; 2 Department of Preventive Medicine, College of Medicine, Korea University, Seoul, South Korea; 3 Institutes for Transdisciplinary Scholarships, University of Calgary, Calgary, Canada; 4 Alliance for Health Policy and Systems Research, World Health Organization, Geneva, Switzerland; 5 Department of Global Public Health, Karolinska Institutet, Stockholm, Sweden; 6 Sachs’ Children and Youth Hospital, Stockholm, Sweden; PLOS: Public Library of Science, UNITED STATES OF AMERICA

## Abstract

Climate change is impacting health, social systems and livelihoods of populations across the world with sub-Saharan Africa being particularly hard-hit. Lived experiences of the impacts of climate change are paramount to understanding how at-risk communities are uniquely impacted and collectively devise response mechanisms. However, there is limited evidence of the community’s lived experiences of these impacts. This study explored the community experiences of the impacts of climate change-induced extreme weather events in Bududa, Kasese, and Moroto districts in Uganda to inform context specific climate mitigation and adaptation interventions. This qualitative study was conducted among purposively selected community members in areas that frequently experienced extreme weather events, notably drought, floods, and/or landslides. The study involved 14 focus group discussions, separate by sex and age. The recorded interviews were audio-recorded, transcribed verbatim, and inductively analyzed according to themes. The analysis revealed seven themes related to the community impacts of climate change and corresponding coping measures. These were: injuries and deaths, infectious disease spread, disrupted livelihoods, food insecurity, poor mental health, difficulties accessing health, and disruption of education services. Communities took various measures to adapt to extreme weather events including environmental conservation and protection, modifying farming practices, diversifying their livelihoods, seasonal migration, seeking health care from alternative sources, and organizing themselves into financial support groups. Floods, landslides, and drought negatively affected the communities with wide-ranging impacts across health and social domains with which they struggled to cope. There were gaps in capacity, equity, system performance, and overall community resilience. As extreme weather events become more frequent, there is a need to strengthen community mitigation and adaptive capacity to enhance systems resilience. Communities should be at the centre of any such efforts.

## Introduction

Across the globe, climate change-induced extreme weather events are increasing in frequency and intensity. In Africa, the global average temperature and sea levels have risen more than the global average since pre-industrial times [1]. These climatic changes are contributing to an increased occurrence, frequency, and intensity of extreme weather events such as heat waves, drought, floods, and cyclones [1]. The impacts of these extreme weather events disproportionately impact sub-Saharan Africa [2,3]. These climate-induced extreme weather events have greatly impacted health, social systems, and livelihoods across the continent, affecting diverse and large populations [4,5]. Extreme weather events have contributed to disease spread and mortality, displacement of populations, food insecurity, environmental degradation, reduced agricultural production, and damage to infrastructure [4,6]. Communities in sub-Saharan Africa are more vulnerable due to their high dependence on subsistence agriculture, which is particularly sensitive to climate conditions. While there have been increasing efforts in documenting the impact of extreme weather events on populations through quantitative investigations, there is a paucity of research that examines the community’s lived experiences of these impacts and their coping mechanisms.

As the impacts of climate change on communities continue to increase, there is an urgent need for mitigation and adaptation strategies. To effectively contribute to this, the perspectives and knowledge of local communities, who are often on the front line of climate change impacts, are paramount. These perspectives can help to understand how communities are uniquely impacted and collectively devise response mechanisms to deal with the climate change impacts [7–10]. Communities are also better placed to share their priorities, needs, knowledge, and capacities, which are key to their empowerment, coping, adaptation, and resilience [9–11]. Documenting community experiences also bolsters community engagement in climate change mitigation and adaptation efforts at the individual and community levels. This study thus explored community experiences of the impacts of climate change-induced extreme weather events in three districts in Uganda hard hit by extreme weather events to inform mitigation and adaptation mechanisms to climate change.

## Materials and methods

### Study area

**Fig 1:**
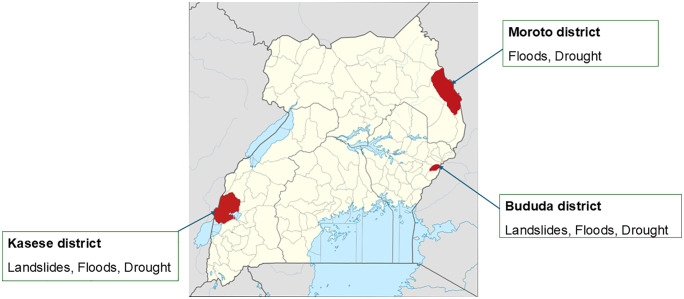
Map of Uganda showing the study districts Attribution: OpenStreetMap contributors, Jarry1250, NordNordWest/Wikipedia.

This study was conducted in Bududa, Kasese and Moroto districts in Eastern, South-western, and mid-Northeastern Uganda respectively (Fig 1). These districts are among those that frequently experience extreme weather events, especially landslides (Bududa, Kasese), and floods and drought (Bududa, Kasese, and Moroto). In 2010, Bududa experienced one of the deadliest landslides worldwide that year, which buried three villages and led to over 365 deaths and over 5000 displacements [12,13]. The district has subsequently experienced recurrent landslides including in 2011, 2012, 2018 and 2019. In Kasese, rainy seasons usually come along with flooding resulting in direct deaths but also damage to infrastructure and population displacement. In 2013, the district experienced one of the worst flooding events submerging nine sub-counties, destroying at least 1000 houses, and displacing 10,000 people [6]. In 2020, over 120,000 people were displaced by floods in Kasese district. Major flooding events have also been registered in 2021 and 2024 Moroto district experiences droughts and prolonged dry spells, strong winds as well as floods from different extreme events impacting agriculture and pastoralism leading to chronic food security and malnutrition [14]. In 2020, the district experienced one of the worst drought years in a decade leading to widespread hunger, livestock deaths, and severe water shortages. Other major events have also been experienced in 2022 and 2023–2024. In the districts, data collection was conducted in sub counties that had been most impacted by extreme weather events.

### Study design and population

We conducted a qualitative descriptive study to explore community experiences of the impacts of climate change-induced extreme weather events. A qualitative descriptive research design focuses on providing a rich and detailed summary of experiences directly from participants’ perspectives. The study used focus group discussions (FGDs) among purposively selected community members in areas that had frequently experienced extreme weather events. Community members were included if they had lived in the area for at least one year while those who were mentally incapacitated were not included.

### Data collection

A total of 14 FGDs were conducted among community members divided by sex (male, female) and age group (18–40 years, 41–65 years) and guided by the principle of data saturation (Table 1). Data saturation occurs when during the process of data collection and/or analysis, no newer information arises with subsequent interviews indicating that further data collection and/analysis is unnecessary. The research team held daily debrief meetings during the data collection period to discuss emerging issues from the FGDs and saturation was considered reached when no new information arose from the meetings. The FGDs were convened at selected public places within the community that offered privacy to the study participants and involved between 8–12 people. The FGD was supported by a guide with questions about the impacts of extreme weather events on the community and their coping mechanisms (S1 File). The FGDs were conducted by research assistants who were both male and female with training in public health and experience conducting qualitative research. Neither the investigators nor the research assistants had any prior relationship with the study participants or other involvement with the study communities. During the discussions, one researcher facilitated the discussion while the other took notes and recorded the session. The FGDs lasted between 80–100 minutes and were conducted in the local languages of the study districts which were: *Lugisu* (Bududa), *Lhukonzo* (Kasese) and *Ngakarimajong* (Moroto) using translated FGD guides. Recruitment of participants for the FGD was done by the community leaders or community health workers in the selected affected communities who identified eligible and available members considering prescribed age range and sex. The FGDs were conducted from 28^th^ February 2024–23^rd^ March 2024 and did not align with any occurrence of extreme weather events within the study districts.

**Table 1:** Focus group discussions conducted.

Sex and age group	Bududa	Kasese	Moroto	Total
**Male**				
18 to 40 years	1	1	1	3
41 to 65 years	1	2	1	4
**Female**				
18 to 40 years	2	1	1	4
41 to 65 years	1	1	1	3
**Total**	5	5	4	14

### Data management and analysis

The discussions were audio-recorded and transcribed verbatim and simultaneously translated into English. The transcripts were checked and verified by another team member while listening to parts of the original audio recordings. Study team members read a few selected transcripts several times and generated a code book. All transcripts were imported into Atlas ti version 7.0 software (GmBH, Berlin) and coding done by two researchers (RN and SK) inductively following the semantic approach, with new codes appropriately added. At the end of the coding process, and guided by thematic analysis [15] , similar codes were grouped into sub-themes which were synthesized into themes. The presentation of the study findings was supported by participant quotations.

### Ethical considerations

This study obtained ethical approval from the Research and Ethics Committee of Makerere University School of Public Health (SPH-2023–520) and was registered by the Uganda National Council for Science and Technology (HS3758ES). All study participants provided written informed consent before participating in the study and also consented for the audio recording. To ensure confidentiality of the study participants, study transcripts were anonymized and access to them restricted to only the study team members.

## Results

### Characteristics of study participants

The study involved 140 participants, aged between 18 and 65 years, and equally distributed between males and females. Most of the participants were farmers and married (Table 2).

**Table 2:** Characteristics of the study participants.

Characteristic	Number of participants
**Sex**	
Male	69
Female	71
**Age group (years)**	
18–40	66
41–65	74
**Education**	
None	50
Primary	60
Secondary and above	30
**Marital status**	
Single	12
Married	125
Widow	3
**Occupation**	
Farming	108
Business	11
Other	21
**Number of children**	
0	12
1 – 5	56
6 - 10	58
11+	14

### Impacts of extreme weather events and community adaptation mechanisms

The analysis revealed seven themes of how climate-induced extreme weather events impacted communities and how they coped. These were injuries and deaths; infectious disease spread; disruption of livelihoods; food insecurity; propagation of poor mental health; difficulties accessing health care; and disruptions to education services as described below with supportive quotations.

### Injuries and deaths

FGD participants reported that landslides and floods were associated with injuries and deaths in the community. They noted that these events trapped or swept away people, leading to injuries including fractures, drowning, and deaths.

“*When the floods occur, even people are swept away. Recently, about four people were swept by floods. One person had gone to the farm and when it rained, one side was flooded, and yet there was a river on the other side. She was trapped and did not make it*.” [Participant 2, FGD (males 41-65 years), Kasese district]

Community members noted that injuries and fractures usually overwhelm health facilities in the aftermath of landslides and floods. Whereas the participants felt everyone was at risk, they noted that children and women were particularly more vulnerable.

“*These extreme events have finished our people. One landslide took five children at once; another took three and then two children*.” [Participant 2, FGD (Females 18-40 years), Kasese district]

To prevent injuries and deaths, community members highlighted seasonal migration to safer areas as an adaptation strategy when they deemed the risk of extreme weather events to be high. Some reported engaging in agroforestry techniques, such as planting trees and digging or desilting trenches, to restore vegetation and divert water from their homes. Others avoided cultivation near rivers to minimize flood impact. Some of these activities were undertaken individually and others through community groups such as the village disaster management committee initiated in high-risk areas or non-governmental organizations (NGOs). Additional coping strategies included using weather forecasts and community alerts to warn of potential floods and landslides, educating communities on floods and landslide risks and preparedness, and relocation of community members to safer areas. The community also relied on available early warning systems to alert others using whistles or megaphones to support quick and effective community action. In Kasese district, some community members also received training from NGOs on how to administer first aid and safely rescue community members trapped by floods.

“*We have a device that tracks water levels in the river and once it starts to flood, the alarm goes off which alerts the community members of potential danger. One organization provided us whistles so we wake up and start whistling such that those near the river can move away*.” [Participant 9, FGD (Male 18-40 years), Kasese district]

### Infectious disease spread

Extreme weather events, especially landslides and floods, were said to lead to the contamination of water sources and destruction of sanitation facilities contributing to the spread of waterborne diseases such as diarrhea, dysentery, typhoid, and cholera. The lack of safe water for drinking and indiscriminate disposal of excreta also increased the risk of waterborne diseases. Flooded areas often became breeding places for mosquitoes increasing malaria transmission. The transmission of lymphatic filariasis was also reported. In the aftermath of the event, sometimes communities are moved to other areas which increases the risk of diseases transmission, including tuberculosis due to overcrowding. Inadequate access to water also linked to poor personal hygiene and related diseases such as trachoma, scabies, and skin infections. Pneumonia, asthma attacks, allergies, and nasal congestion were noted to be high among children during extreme cold weather. Overall, children were reported to be the most at risk of infectious diseases.

“*When landslides occur, the latrines are destroyed, and the fecal matter contaminates our drinking water spreading waterborne diseases. Also, muddy waters from the mountains settle at the bottom here, and act as breeding grounds for mosquitoes transmitting malaria*.” [Participant 7, FGD (Female 18-40 years), Bududa district]

Drought was reported to lead to reduced access to water, forcing communities to share limited sources with animals and increasing the risk of contamination. Drought was also associated with increased coughs as dry air irritated the throat and airways, affecting all age groups.

“*Drought dries up water sources and you are forced to fetch water from where the cows drink it. It is the same water that we use for drinking with our children leading to disease transmission. Another thing, during drought, when the wind blows, it comes with flu and cough affecting us.*” [Participant 2, FGD (Males, 18-40 years), Kasese district]

The community reported that they were regularly sensitized about infectious disease prevention by the authorities. Other reported preventive measures were ensuring access to safe water, improving sanitation and hygiene, and clearing stagnant water from the vicinity of households.

### Disruption of livelihoods

Landslides and floods were highlighted to contribute to the destruction of housing and other personal belongings that greatly impacted communities, displacing and forcing them away from their traditional home areas. These events were also linked with led to soil degradation and erosion which destroyed farmland – especially the fertile topsoil–, crops, and animals further disrupting community livelihoods. In the study districts, crop farming, animal rearing, and pastoralism (for Moroto) were the main sources of food and income for households. Additionally, droughts led to drying up of crops and water sources for animals, and reduced water availability for farming and household use. Animal illness and deaths due to drought-induced harsh conditions were also reported. Other times, rain was noted to come at unexpected times, disrupting planting and harvesting cycles severely reducing crop yields. Changing weather patterns also increased pests and diseases affecting yields. Community members reported that the disruption of their livelihoods impacted their income levels and increased the risk of poverty in the community. The reduced access to food and rising expenditure on it exacerbated the problem with families having less to spend on other services.

**“***We are stuck in poverty because we use the money that we borrow from the bank to invest in crop farming and so when it doesn’t yield because of drought we are hugely impacted. Because of this, children fail to go to school that’s why you see the big population here with few educated children*.” [Participant 3, FGD (Males 41-65 years), Kasese district]

In some areas, community members further destroyed the environment to obtain firewood or burnt charcoal that they sold to provide for their families. Engaging in petty trade was also reported as another form of livelihood diversification. Disruptions to livelihoods were also said to increase pressures, especially on young girls, to engage in unhealthy sexual relationships as voiced in the older women FGD who were concerned about its impact on adolescents. For the men, engagement in crime, especially theft of food and other items due to the lack of economic opportunities, was reported in their groups. The situation was reported to be worse among women and men who relocated to other areas, especially those living in camps.

“*We have become poor with no food to feed our families. When the poverty is extreme, you find our girls being lured into early sexual relationships by men in exchange for money or gifts with various risks*.” [Participant 9, FGD (Females 41–65 years), Kasese district)

### Food insecurity

Drought, landslides, and floods were associated with food insecurity in the community. Landslides and floods destroyed large farmlands, killed animals, and contributed to food insecurity in their aftermath. Even when communities were relocated, they did not have sufficient food resources or farmland to grow additional food. Drought dried up the crops and limited food and water options for animals leading to their death. The major consequences of food insecurity reported by the community were unfulfilled nutritional needs leading to malnutrition, especially among children. Those on long-term medication such as tuberculosis or brucellosis patients, who needed to take medication after meals reported impacts on their adherence to treatment. Community members also reported that ulcerative conditions were on the rise due to irregular eating habits.

“*People suffering from tuberculosis need food before they take their medication, but when there is nothing to eat, they stop taking their medicines. This makes it difficult to fight the disease in our community. Some TB patients do not go for their medicines for even up to two months.*” [Participant 11, FGD (Females 18-40 years), Moroto district]

To adapt, community members grew drought-resistant crops, especially those that required less water and yielded faster or adjusted planting seasons to match new rainfall patterns. In Kasese district, local organizations mobilized community members to store food during bumper harvests which were resorted to during periods of food insecurity. Rainwater harvesting and construction of small ponds were also reported. Some communities used small scale irrigation to support vegetable growing to improve their diet and reduce malnutrition. However, this was also affected by drought and the yield failed or was low. In extreme circumstances, some NGOs provided food to the community during periods of food scarcity.

“We have been supported by an NGO to grow and water vegetables during the dry season to improve our diet and reduce malnutrition. Sometimes we get good vegetables. Other times when the drought is extreme, we fail to harvest anything.” [Participant 4, FGD (Males, 18-40 years), Moroto district]

### Propagation of poor mental health

Extreme weather events negatively impacted the community’s mental health. Community members noted being very anxious and worried about the potential of another event occurring at catastrophic levels, especially for landslides and floods. The other contributing factors to their mental health predicament were the loss of dear ones, livelihoods, crops, animals, housing and harsh living conditions. Some members reported that the pressure on men to provide for their families amidst the difficult circumstances led them to be violent towards their women, increasing household disharmony and separation.

“*Following landslides, community members are always worried they will recur. Whenever it threatens to rain, people wake up and sit at night because of fear of being ambushed by landslides. Another worry is that when the landslides hit their dwelling places, they don’t know where else to run for refuge. These worries have caused mental health problems to some of the community living here*.” [Participant 2, FGD (Females, 18-40 years), Bududa district]

Community members noted that the increased stress and anxiety induced by extreme weather events also predisposed them to hypertension and some reportedly had it. Other community members noted unhealthy coping strategies including excessive alcohol consumption. Positively, community members organized themselves into village savings and loan groups, pooling resources together that can be lent to others during times of need. Community members noted that access to emergency financing reduced their worries and anxiety and improved social connectedness.

“*We have our own associations, each comprising 15 to 20 members, where we make our humble financials contributions. We deposit our savings from which we also lend amongst ourselves, especially during emergencies including illness. The fund reduces our worries about finances*.” [Participant 4, FGD (Males 41-65 years), Bududa district]

### Difficulties accessing health care services

Infrastructure damage from landslides and floods, such as destroyed roads, bridges, and health centers disrupted access to healthcare. This made it difficult for community members to reach health facilities and for medical supplies and health workers to access remote areas. At other times, the community had to travel far longer distances to access care from functional facilities that were further away. These challenges led to delayed health care seeking or a quest for alternative informal care such as from traditional health practitioners. Yet, the extreme weather events were accompanied by increased demand for health services including those that required urgent care.

“*The* *last time this area was struck by landslides, the roads and bridges were destroyed which reduced the flow of patients to health facilities. The destroyed roads also reduced the rate at which health workers were reporting to facilities. The vehicles bringing medical supplies also found it hard to reach facilities. So, whenever the floods come, the community is worried.*” [Participant 8, FGD (Females, 18-40 years), Bududa district]

While at the health facility, community members reported the number of patients accessing health care at the few functional facilities to be overwhelming, leading to overcrowding and long waiting times. The health workers were sometimes unavailable, or reportedly overwhelmed by the clients and drug shortages were commonplace. The referral system was also impacted by difficulties moving from one facility level to the other, which were far, yet most facilities did not have ambulances. This exacerbated the health conditions or led to deaths.

“*Our two health centers reach a point where they cannot effectively provide services to the community because the number of patients who want treatment due to injuries caused by landslides is high. So, because these patients have tried their best to find their way to the facilities, the few health workers available are overwhelmed that some injured patients end up not being attended to*.” [Participant 1, FGD (Females 18-40 years), Bududa district]

Districts were reported to have emergency treatment centers or mobile or satellite health units for affected areas where communities could access care when their usual access is hampered by extreme weather events. Health centers were also stocked with medicines for common illnesses to prepare for the expected increased demand during drought or floods. Community members also reported sensitizations by NGO actors on disease prevention and nutrition in advance of extreme weather events. Community health workers were also deployed to assist health facility personnel by providing basic treatment, especially for children in remote villages, reducing travel distances. For maternity-related care, some community members accessed it from traditional birth attendants when they could not access health facilities. Through their community groups, community members reported fundraising or directing their funds to support medical expenses for one another. To deal with transport difficulties and destroyed infrastructure, the community also sometimes used stretchers to transport sick members to the health facility.

“*We have devised wooden stretchers that we use to transport sick community members to the facility especially when the roads have been damaged and we cannot have ambulance access. With stretchers, we can use small paths and still make it to the health facility*.” [Participant 7, FGD (Males 18-40 years), Kasese district]

### Disruption of education services

Community members noted that landslides and floods destroyed schools. Other times, schools were used as shelters for temporary accommodation of community members. The destruction of school buildings, roads, and bridges also forced schools to close with children unable to access them. The destruction of schools and disruptions to schooling was noted to lead to reduced academic performance, attendance, and increased school dropouts. Another factor that impacted school attendance was the increased incidence of infectious diseases among children who then lost school days during treatment and recovery. The negative impacts on the education performance of schools were also noted to be long term beyond the extreme weather events.

“*Our primary school was destroyed by the landslides, and its academic performance has since deteriorated. The school population has also since reduced*.” [Participant 2, FGD (Females 18-40 years), Bududa district]

Some community members especially young males reported supporting the repair of roads and bridges after extreme weather events to ease movement to schools and health facilities. In one area, the youths had made a makeshift bridge for this purpose.

“*As a community we made a makeshift bridge connecting our village to the trading centre using tree trunks that we collected by ourselves. This has made it easy for the children to cross over even when it floods to go to school*.” [Participant 10, FGD (Males 18-40 years), Bududa district]

## Discussion

This study explored the lived experiences of communities of the impacts of climate change-induced extreme weather events in three districts in Uganda. Floods, droughts, and landslides greatly impacted the lives of communities through various pathways. Community members reported injuries and deaths, infectious disease spread, disrupted livelihoods, food insecurity, poor mental health, difficulties accessing health services, and disruption to education services. To adapt, the community employed various strategies, which though crude, were largely positive. The research revealed gaps in capacity, equity, and system performance and overall community resilience that require urgent attention.

The occurrence of rapid onset events such as floods and landslides is associated with injuries and deaths [16,17] with children, women, the elderly, and those living with disabilities most vulnerable. These groups have been consistently highlighted in the climate change literature as disproportionately affected due to physical, economic, power, and cultural disadvantages [17–21]. Previous events in the country, including in the study districts, recorded several deaths and injuries, greatly impacting communities [6,12,13,22,23]. One study reported that injury rates were higher and more severe among flood-affected than landslide-affected areas and among individuals below 42 years [22]. Injuries and deaths are likely due to ineffective early warning systems, attributed to factors such as deficiencies in technology, inadequate financing, social-cultural constraints, and communication challenges [24]. The effectiveness of the early warning systems could also be influenced by the changing and unpredictable weather patterns coupled with the occasional resistance of communities to relocate to safer areas. In some cases, community members expressed dismay at leaving their land which they deem to be fertile and productive to migrate to areas without equivalent access to resources, especially land to grow crops or rear animals. In the study, community members who had been relocated away from their homelands found it much harder to obtain and sustain their livelihoods. Recent research in the same community has highlighted that unfavourable climate, disputes over land, and poor social services, and housing conditions contribute to the situation [25]. In this study, the consequences related to poorer livelihoods included crime, unhealthy sexual relationships, household disharmony, and violence. Rigorous and proactive planning and building community trust is important for effective and equitable relocation [26,27]. Overall, effective early warning systems and strengthening community response mechanisms could go a long way in reducing injuries and deaths from extreme weather events. A recent review reported that flood early warning systems were effective in reducing injuries and deaths in addition to other benefits including enhancing food security and reducing property damage [28]. However, the effectiveness of such systems are buttressed by the role of the community in understanding, interpreting, disseminating, and responding to the events [28].

The community members reported difficulties accessing food and looking after their households. In a previous study in Bududa and Kasese districts, resettled communities noted challenges in accessing food which discouraged people from leaving [25,29]. Even when relocated, some community members usually go back to their original areas deemed risky citing poverty and population pressures [29]. In another study in Nepal, landslide affected communities indicated that livelihood indices such as food, shelter, and employment were most deficient [16]. In Iran, communities affected by drought reported several impacts to their health, livelihoods and social cohesion noting that it propagated migration [30]. Community needs should be holistically examined and catered for, including aspects of livelihood provisions and continuity, when they are relocated from their home areas. Due to the destruction of property, crops, and animals that are reported in the affected communities, the community equally requires support to reestablish their livelihoods. The disruption of livelihoods and/or the destruction due to extreme weather events contributed to the high food insecurity that was reported in all study districts, especially in Moroto that experienced extreme drought. The district also registered higher levels of malnutrition. Community adaptation mechanisms that involve food storage during bumper harvests as reported in Kasese district are critical in dealing with the situation in addition to promoting food preservation and water storage. Overall, policy and programmatic efforts are required to support communities to build resilience in their livelihoods. A study among vulnerable communities in Uganda’s Elgon region identified several coping mechanisms for landslides and floods, including the adoption of improved farming practices, livelihood diversification, external support, and the use of indigenous knowledge for weather forecasting and preparedness [29]. Pacific Island communities have been exemplary in building community resilience to cyclones and drought through particular planting techniques, innovative water storage, and food preservation practices [9]. Such strategies would also help to alleviate food insecurity due to the extreme weather events.

Extreme weather events were linked to significant health impacts as described by the communities, especially an increase in waterborne infectious diseases, similar to other studies [6,31,32]. This was mostly attributed to the dire water, sanitation and hygiene situation which is a considerable concern when extreme weather events occur [13,33]. High risk communities ought to be supported and adequately prepared to deal with these potential eventualities including increasing awareness of the risk of waterborne diseases. During drought, dust storms can increase air pollution exacerbating respiratory conditions such as asthma [34], which were also reported. Experiencing extreme weather events, the subsequent displacement, and loss of lives and livelihoods was associated with fear and anxiety that affected the mental health of affected communities. Increased stress, anxiety, depression, and post-traumatic stress disorder and other mental health disorders are common among those affected by such events [35,36]. The pathways include disruption of conditions that support mental health, compounding of existing stressors, and psychological distress due to climate inaction [37,38]. In Indonesia, the mental wellbeing of landslide-displaced populations was interconnected with their livelihoods and connection to land and community [30,38]. In Ghana, flood-affected communities reported getting stressed with the slightest possibility of rains [17]. Psychosocial services need to be provided for affected communities to enable them to navigate the aftermath of experiencing extreme weather events. Community support structures such as social groups and voluntary savings groups were reported, and such mechanisms ought to be strengthened. Generally, community engagement in adaptation strategies through education, awareness campaigns, and participatory approaches can empower individuals to protect their health and contribute to resilience efforts [8,39]. Overall, there is urgent need to bolster the psychosocial dimension of resilience and for provision of integrated services to support psychological coping and well-being.

Climate-induced extreme weather events also disrupted services access especially health and education services through destroying roads, bridges, and health facilities and schools. The increased demand for health services in the aftermath of the event also compounded the situation, overwhelming health workers and facilities. Several studies have documented the impact of extreme weather events on the health system including damaging infrastructure, drugs, and information systems and disrupting services delivery [40,41]. Extreme weather events also lead to school closures, halt learning, and negatively impact education outcomes [42]. It is imperative to enhance the resilience of health and education systems to mitigate disruptions in service access during climate-induced extreme weather events. Owing to existing gaps in community resilience at the community level, community members adopted crude strategies to deal with the impacts of extreme weather events such as accessing care from traditional practitioners. Uganda recently launched its Health National Adaptation Plan [43] which aims to strengthen the adaptative capacity of the county’s health system to climate change and its hazards highlighting potential strategies across the ten components of the World Health Organization’s Operational framework for climate resilient and low carbon health systems [7]. The plan is comprehensive, covering multiple interventions including developing guidelines, training and capacity building, and monitoring and evaluating adaptation interventions, and if effectively implemented, could go a long way to strengthening the resilience of the health system to extreme weather events. In addition, clear coordination of efforts, integration of the plan with other climate and health policies and government development plans, and enhanced collaboration between government and NGO actors with local stakeholders are needed.

The community undertook several measures to cope with the extreme weather events they faced ranging from environmental conservation and protection, livelihood diversification, seasonal migration, and organizing into financial saving groups. Most of these measures have been previously reported [9,25,29]. These coping mechanisms were sometimes community-led or supported by the district authorities or other organizations. Districts have, however, expressed concern over limited financing to support adaptation measures overly relying on NGO activities. Even when districts are required to have a small proportion of the budget go towards climate adaptation activities, this is usually deprioritized during implementation yet channeling such resources towards environmental conservation and protection activities could be impactful. Community financial saving groups emerged as a key lifeline to alleviate financial pressures. In several African countries, studies have demonstrated the role of saving groups in increasing financial access, improving livelihoods, and strengthening social cohesion [44,45] and they hold potential in building financial resilience among communities. These groups can also be used for skilling community members, disseminating information, and promoting health. Overall, these community efforts demonstrate initiative which was mostly reactive but shows promise for future climate change interventions. There were also aspects of negative coping including alcohol consumption, crime, and unhealthy sexual relationships that require adequate attention in affected communities. Whereas the community-led adaptation was a strength, it was also a demonstration system failure that pushed community members to fill the gaps [46]. As extreme weather events become frequent, there is need to build both the mitigation and adaptative capacity with a clear and pragmatic role of communities towards systems resilience.

This study delved deeper into the community perspectives of the impacts of climate-change events which is usually not given much attention. The FGDs were also homogeneous, ensuring comfortable and detailed interactions about shared experiences among community members. We also conducted double translation to ensure accuracy and reliability of the translated study tools. Nevertheless, the study could have benefitted from other local stakeholders’ opinions that could have been triangulated with the findings. Also, as several organizations have been supporting the affected communities, some community members may have provided information with the expectation of receiving additional support, however, clarity was made at the beginning and during the interviews that information was only for research purposes. Sampling and self-report bias were also possible in this study.

## Conclusions

Landslides, floods, and drought impacted the community through causing injuries and deaths, increasing the spread of infectious disease, disrupting livelihoods, propagating food insecurity and poor mental health outcomes. Moreover, following the extreme weather events, communities experienced difficulties accessing health and education services. Communities took various measures to adapt to extreme weather events including environmental conservation and protection, modifying farming practices, diversifying livelihoods, seasonal migration, seeking health care from alternative sources, and organizing themselves into financial support groups. Building on these lessons, government together with other stakeholders should strengthen community-based early warning systems, enhance health system resilience, or integrate livelihood improvement and diversification interventions in vulnerable and affected communities. Overall, this study revealed gaps in capacity, equity, and system performance and overall community resilience that require urgent attention. As extreme weather events become more frequent, there is need to strengthen both the mitigation and adaptative capacity with a clear and central role of communities towards systems resilience.

## Supporting information

S1 File Focus group discussion guide for community members. (DOCX)

S1 Checklist COREQ (Consolidated criteria for reporting qualitative studies) checklist. (DOC)
