# Supplementary material for: Community experiences of the impacts of climate-induced extreme weather events in Uganda: A qualitative study
Source: PLOS Glob Public Health. 2026 Mar 20;6(3):e0005887. doi: 10.1371/journal.pgph.0005887 (PMC13004317; doi:10.1371/journal.pgph.0005887)
Supplement: S1 File — (DOCX) [file pgph.0005887.s001.docx]

## **Focus group discussion guide for community members**

1. What are the usual extreme weather events that your community experiences?
2. How do these extreme weather events impact health and healthcare access in your community?

- What impact do these events have on the disease burden of the area? Which diseases tend to occur more often during these extreme weather events? Who are the most affected by these diseases? (*Note: explore separately per extreme weather event mentioned including community adaptation mechanism*)
- How do these events impact your access to health services and who is most impacted? (*Note: explore separately per extreme weather event mentioned including community adaptation mechanism*)

1. What mitigation and adaptation mechanisms do the health facilities employ to deal with the adverse effects of extreme weather events?

- What is done at the district and community levels and by whom?
- What support is provided to the community to increase resilience? What role does the community play?
- What programmes are implemented at the community level to increase health system resilience? (What does the programme do and who funds it?)

1. To what extent are you as the community involved in climate change-related activities in the community?
2. What recommendations do you suggest to support building community and health systems that can deal with the adverse effects of extreme weather events in your community / district?

- What should the ministry of health and other stakeholders do differently?
- What do you need to do differently as the community?
